# A Proposed Method for Solving Fuzzy System of Linear Equations

**DOI:** 10.1155/2014/782093

**Published:** 2014-08-19

**Authors:** Reza Kargar, Tofigh Allahviranloo, Mohsen Rostami-Malkhalifeh, Gholam Reza Jahanshaloo

**Affiliations:** Department of Mathematics, Science and Research Branch, Islamic Azad University, Tehran 18558-17556, Iran

## Abstract

This paper proposes a new method for solving fuzzy system of linear equations with crisp coefficients matrix and fuzzy or interval right hand side. Some conditions for the existence of a fuzzy or interval solution of *m* × *n* linear system are derived and also a practical algorithm is introduced in detail. The method is based on linear programming problem. Finally the applicability of the proposed method is illustrated by some numerical examples.

## 1. Introduction

Systems of simulations linear equations play a major role in various areas such as mathematics, physics, statistics, engineering, and social sciences. Since in many applications at least some of the system's parameters and measurements are represented by fuzzy rather than crisp numbers, it is important to develop mathematical models and numerical procedures that would appropriately treat general fuzzy linear systems and solve them [1]. A general model for solving an *m* × *n* fuzzy system of linear equation (FSLE) whose coefficients' matrix is crisp and right hand side column is an arbitrary fuzzy number vector was first proposed by Friedman et al. [1]. Different authors [2–5] have investigated numerical methods for solving such FSLE. Most of mentioned methods in different articles are based on numerical methods such as matrices decomposition and iterative solutions. Previously mentioned papers do not discuss a lot the possibility of solutions. In addition they cannot find alternative solutions. But the proposed method does not include these defects. Allahviranloo et al. [6] have presented that the above-mentioned method is not applicable and does not have solution generally. This paper sets out to investigate the solution of the fuzzy linear system using a linear programming method. Also we are going to explain the necessary and sufficient conditions for existence of the solutions. The idea of this method can join some uses of linear programming to solve the problems of interval data in [7–9]. The structure of this paper is organized as follows.

In Section 2, we provide some basic definitions and results which will be used later.

In Section 3, we prove some theorems which are used for proposed method and present a practical procedure. The introduced method is illustrated by solving some examples in Section 4 and conclusions are drawn in Section 5.

## 2. Preliminaries

In this section some basic definitions and concepts are brought.

**Definition 1 (by [10]).**

The triangular fuzzy numbers (TFN) are very popular and are denoted by $\tilde{u}=(\alpha ,c,\beta )$ and defined by

(1) $$\begin{array}{c} \tilde{u}=\{\begin{array}{cc} \frac{x-\alpha }{c-\alpha }, & \alpha \leq x\leq c,\\ \frac{\beta -x}{\beta -c}, & c\leq x\leq \beta ,\\ 0, & \text{otherwise}. \end{array} \end{array}$$

*Note*. If *α* = *c*, then TFN is defined:

(2) $$\begin{array}{c} \tilde{u}=\{\begin{array}{cc} \frac{\beta -x}{\beta -\alpha }, & \alpha \leq x\leq \beta ,\\ 0, & \text{otherwise}, \end{array} \end{array}$$

if *β* = *c*, then TFN is defined:

(3) $$\begin{array}{c} \tilde{u}=\{\begin{array}{cc} \frac{x-\alpha }{\beta -\alpha }, & \alpha \leq x\leq \beta ,\\ 0, & \text{otherwise}, \end{array} \end{array}$$

and finally if *α* = *c* = *β*, then TFN is defined:

(4) $$\begin{array}{c} \tilde{u}=\{\begin{array}{cc} 1, & x=\beta ,\\ 0, & \text{otherwise}. \end{array} \end{array}$$

**Definition 2 (by [10]).**

Let $\tilde{u}=(\alpha ,c,\beta )$ be a triangular fuzzy number; then one defines

(5) $$\begin{array}{c} \mathrm{supp}\left(\tilde{u}\right)=\left[\alpha ,\beta \right],\;\;\text{core}\left(\tilde{u}\right)=c. \end{array}$$

**Lemma 3 (by [10]).**

For arbitrary interval $\left[\underline{x},\bar{x}\right]$, $\left[\underline{y},\bar{y}\right]$ the following properties hold:

(i) $\left[\underline{x},\bar{x}]+[\underline{y},\bar{y}]=[\underline{x}+\underline{y},\bar{x}+\bar{y}\right]$,
(ii) $\left[\underline{x},\bar{x}]-[\underline{y},\bar{y}]=[\underline{x}-\bar{y},\bar{x}-\underline{y}\right]$,
(iii) for each *k* ∈ *R*,
  (6) $$\begin{array}{c} k\left[\underline{x},\bar{x}\right]=\{\begin{array}{cc} \left[k\underline{x},k\bar{x}\right], & if\;k\geq 0,\\ \left[k\bar{x},k\underline{x}\right], & if\;k<0. \end{array} \end{array}$$

**Proof**

See [10].

**Definition 4 (by [5]).**

Let *F*(*R*) be a set of all fuzzy numbers on *r* and *I*(*R*) a set of intervals on *R*. The *m* × *n* linear system of equations is as follows:

(7) a11x1+a12x2+⋯+a1nxn=y1,a21x1+a22x2+⋯+a2nxn=y2,⋮am1x1+am2x2+⋯+amnxn=ym,

where the coefficient matrix *A* = (*a*_*ij*_), 1 ≤ *i* ≤ *m*, 1 ≤ *j* ≤ *n*, is a crisp *m* × *n* matrix and *y*_*i*_ ∈ *F*(*R*)⋃*I*(*R*), 1 ≤ *i* ≤ *m*, is called a FSLE.

**Theorem 5 .**

If $\tilde{u}$ and $\tilde{v}$ are triangular fuzzy numbers and if *t* ∈ *R*, then

(i) $\tilde{u}=\tilde{v}$ if and only if $\mathrm{supp}\;(\tilde{u})=\mathrm{supp}\;(\tilde{v})$ and $\text{ core }(\tilde{u})=\text{ core }(\tilde{v})$,
(ii) (8) $$\begin{array}{c} \mathrm{supp}\;\left(t\tilde{u}+\tilde{v}\right)=t\;\mathrm{supp}\;\left(\tilde{u}\right)+\mathrm{supp}\;\left(\tilde{v}\right), \end{array}$$
(iii) (9) $$\begin{array}{c} \text{ core }\;\left(t\tilde{u}+\tilde{v}\right)=t\;\text{ core }\left(\tilde{u}\right)+\text{ core }\left(\tilde{v}\right). \end{array}$$

**Proof**

See [10].

**Theorem 6 .**

Let *A* be *m* × *n* matrix and *b* an m-vector. The system *Ax* = *b* with condition *m* ≤ *x* ≤ *M* has a solution if and only if the optimal solution of the below system is zero:

(10) Min⁡ z=1xa
s.t.Ax+xa=b,m≤x≤M,xa≥0.

**Proof**

See [11].

## 3. Proposed Method

First of all the following definitions and theorems are introduced.

**Definition 7 .**

Let $I=[\underline{x},\bar{x}]$; it can be written as *I* = [−*L*_*I*_, *L*_*I*_]+[*M*_*I*_, *M*_*I*_] such that

(11) $$\begin{array}{c} L_{I}=\frac{\bar{x}-\underline{x}}{2},\;\;M_{I}=\frac{\bar{x}+\underline{x}}{2}. \end{array}$$

Now one can have the following theorems.

**Theorem 8 .**

Let $I=[\underline{x},\bar{x}]$ and $J=[\underline{y},\bar{y}]$ be intervals and *k* ∈ *R*; then

- *I* = *J* if and only if *L*_*I*_ = *L*_*J*_ and *M*_*I*_ = *M*_*J*_,
- if $I+J=[\underline{x},\bar{x}]+[\underline{y},\bar{y}]$, then *L*_*I*+*J*_ = *L*_*I*_ + *L*_*J*_ and *M*_*I*+*J*_ = *M*_*I*_ + *M*_*J*_,
- let *S* = *kI* such that *k* ∈ **R**; then *L*_*S*_ = |*k* | *L*_*I*_ and *M*_*S*_ = *kM*_*I*_.

**Proof**

Proofs of parts (i) and (ii) are obvious so we prove part (iii). Let *k* < 0 such that

(12) $$\begin{array}{c} S=k\left[\underline{x},\bar{x}\right]=\left[k\bar{x},k\underline{x}\right]=\left(\left[-L_{S},L_{S}\right]+\left[M_{S},M_{S}\right]\right), \end{array}$$

where

(13) $$\begin{array}{c} L_{S}=\frac{k\underline{x}-k\bar{x}}{2}=-k\frac{\bar{x}-\underline{x}}{2}=\left|k\right|L_{I},\\ M_{S}=\frac{k\underline{x}+k\bar{x}}{2}=k\frac{\bar{x}+\underline{x}}{2}=kM_{I} \end{array}$$

for *k* ≥ 0 the proof is the same.

**Theorem 9 .**

If *A* is a *m* × *n* matrix and *X* is an interval vector, then

(14) $$\begin{array}{c} AX=\left[-A^{+}L_{X},A^{+}L_{X}\right]+\left[AM_{X},AM_{X}\right], \end{array}$$

where *A*_*ij*_^+^ = |*A*_*ij*_| and $L_{X_{j}}=\left(\bar{X_{j}}-\underline{X_{j}}\right)/2$ and $M_{X_{j}}=\left(\bar{X_{j}}+\underline{X_{j}}\right)/2$.

**Proof**

According to Theorem 8, for *i* = 1,2,…, *m*, we have

(15) AiX=∑j=1j=nAij[Xj_,Xj¯]=∑j=1j=nAij([−LXj,LXj]+[MXj,MXj])=∑j=1j=n|Aij|[−LXj,LXj]+Aij[MXj,MXj]=∑j=1j=n|Aij|[−LXj,LXj]+∑j=1j=nAij[MXj,MXj]=[−A+iLX,A+iLX]+[AiMX,AiMX].

**Theorem 10 .**

If *A* is a *m* × *n* matrix and *X*, *b* are two interval vectors, then the system *AX* = *b* has solution(s) if and only if the following systems have solution:

(16) $$\begin{array}{c} A^{+}L_{X}=L_{b},\;L_{X}\geq 0,\\ AM_{X}=M_{b}. \end{array}$$

**Proof**

Let *X* be an interval solution of *AX* = *b*; then according to Definition 7 *X* = [−*L*_*X*_, *L*_*X*_]+[*M*_*X*_, *M*_*X*_] with *L*_*X*_ ≥ 0 and according to Theorem 9, we have

(17) $$\begin{array}{c} AX=\left[-A^{+}L_{X},A^{+}L_{X}\right]+\left[AM_{X},AM_{X}\right] \end{array}$$

but *AX* = *b* and by using part (i) of Theorem 8 this means

(18) $$\begin{array}{c} A^{+}L_{X}=L_{b},\;L_{X}\geq 0,\\ AM_{X}=M_{b}; \end{array}$$

the converse holds obviously.

**Theorem 11 .**

The system *A* ^+^ *L*_*X*_ = *L*_*b*_ with condition *L*_*X*_ ≥ 0 has a solution if and only if optimized value of the below linear programing is zero:

(19) Min⁡ z=1xa
s.t.A+LX+xa=Lb,ŁX,xa≥0.

**Proof**

This is proved by using Theorem 6.

Now we are going to apply the same method for solving $A\tilde{X}=\tilde{B}$, where $\tilde{B_{i}}$ is TFN.

**Theorem 12 .**

If *A* is a *m* × *n* matrix with crisp coefficients and $\tilde{B}$ is a TFN vector the same as $\tilde{B_{i}}=(\alpha_{i},c_{i},\beta_{i})$, then the system $A\tilde{X}=\tilde{B}$ has TFN solution(s), $\tilde{X}$, the same as $\tilde{X_{i}}=(\underline{x_{i}},x_{i},\bar{x_{i}})$ if and only if the systems (20) have solution:

(20) $$\begin{array}{c} A\left(S_{\tilde{X}}\right)=S_{\tilde{B}},\\ A\left(C_{\tilde{X}}\right)=C_{\tilde{B}},\\ C_{\tilde{X_{j}}}\in \mathrm{supp}\left(\tilde{X_{j}}\right)\;j=1,2,\ldots ,n, \end{array}$$

where

(21) SBi~=supp⁡(Bi~)=[αi,βi], i=1,…,m,SXj~=supp⁡(Xj~)=[xj¯,xj_], j=1,…,n,CBi~=core(Bi~)=ci, i=1,…,m,CXj~=core(Xj~)=xj, j=1,…,n.

**Proof**

From part (i) of Theorem 5 $\tilde{X}$ is a TFN solution of system $A\tilde{X}=\tilde{B}$ if and only if

(22) $$\begin{array}{c} \mathrm{supp}\left(A^{i}\tilde{X}\right)=\mathrm{supp}\left(\tilde{B_{i}}\right),\;i=1,2,\ldots ,m,\\ \text{core}\left(A^{i}\tilde{X}\right)=\text{core}\left(\tilde{B_{i}}\right),\;i=1,2,\ldots ,m. \end{array}$$

So according to part (ii) of Theorem 5 for *i* = 1,2,…, *m* we have

(23) SBi~=supp⁡(Bi~)=supp⁡(AiX~)=supp⁡(∑j=1j=nAijXj~)=∑j=1j=nAijsupp⁡(Xj~)=AiSX~.

Also according to part (iii) of Theorem 5 for *i* = 1,2,…, *m* we have

(24) CBi~=C(Bi~)=C(AiX~)=C(∑j=1j=nAijXj~)=∑j=1j=nAijC(Xj~)=AiCX~

but $C_{\tilde{X_{j}}}\in \mathrm{supp}\left(\tilde{X_{j}}\right)$, *j* = 1,2,…, *n*.

**Theorem 13 .**

The system $AC_{\tilde{X}}=C_{\tilde{B}}$ with condition $C_{\tilde{X_{i}}}\in \mathrm{supp}\;(\tilde{X_{i}})$ has a solution if and only if optimal solution of the following linear programing is zero:

(25) Min⁡ 1xa
s.t.ACX~+xa=CB~,CXi~∈supp⁡(Xi~),xa≥0.

**Proof**

This is proved by using Theorem 6.

## 4. Numerical Example

Here we describe the proposed method completely and step by step by two examples. In the first example, system is introduced in which matrix *A* is a definite (crisp) matrix and *B* is a vector of triangular fuzzy numbers and a solution is then calculated for it. In the second example, an interval system without solution is outlined.

**Example 1 .**

Consider the following 4 × 6 fuzzy system in which $\tilde{X}$ is a triangular fuzzy vector:

(26) −2X1~+4X2~−3X3~−5X4~−2X6~=(−1,0,1)+4X2~−6X3~−2X4~+6X5~−4X6~=(4,5,8)5X1~−2X3~−3X4~+8X5~+3X6~=(−5,−3,−1)−9X1~+1X2~+8X3~−8X4~+4X5~+7X6~=(0,2,5).

To solve this system, we proceed in two successive stages according to Theorem 12.

**Stage 1 .**

Find $\text{Supp}\;(\tilde{X})$, where $\text{Supp}\;(\tilde{X_{j}})$ is the interval $\left[\underline{x_{j}},\bar{x_{j}}\right]$. Therefore, the following system must be solved:

(27) [−24−3−50−204−6−26−450−2−383−918−847][[x1_,x1¯][x1_,x1¯][x1_,x1¯][x1_,x1¯][x1_,x1¯][x1_,x1¯]]  =[[−1,1][4,8][−5,−1][0,5]].

**Stage 2 .**

After calculating the intervals $\left[\underline{x_{j}},\bar{x_{j}}\right]$ in the first stage, search for $\text{Core}(\tilde{X_{j}})=x_{j}$ that satisfies $\underline{x_{j}}\leq x_{j}\leq \bar{x_{j}}$. Therefore, the following system must be solved:

(28) [−24−3−50−204−6−26−450−2−383−918−847][x1x2x3x4x5x6]=[05−32],xj_≤xj≤xj¯, j=1,2,…,6.

Here, the system of the first stage (i.e., system (27)) is solved. It is an interval system, so it must be solved in two substages according to Theorem 10. The* first substage* is finding the interval length, that is, *L*_*X*_. Thus, the following system is solved:

(29) [243502046264502383918847][LX1~LX2~LX3~LX4~LX5~LX6~]=[1622.5]0≤LXj~ j=1,2,…,6.

Here right hand side is $L_{\text{Supp}(\tilde{B})}$ and $L_{\tilde{X_{j}}}=\left(\left(\bar{x_{j}}-\underline{x_{j}}\right)/2\right)$. But according to Theorem 11, the presence of solution (29) is equivalent to the following LP problem:

(30) Z∗=min⁡ xa1+xa2+xa3+xa4s.t.[243502046264502383918847][LX1~LX2~LX3~LX4~LX5~LX6~]  +[xa1xa2xa3xa4]=[1622.5], 0≤LXj~ j=1,2,…,6, 0≤xai~ i=1,2,…,4.

We solve it with the* Simplex* method [11]. Since the optimal value (Z*) is zero, system (29) has the following solution:

(31) $$\begin{array}{c} L_{\tilde{X}}={\left[\begin{array}{cccccc} 0.03315, & 0.06970, & 0.05475, & 0.0714, & 0.16375, & 0.0668 \end{array}\right]}^{t}. \end{array}$$

The* second substage* is to find the center of $\left[\underline{x_{j}},\bar{x_{j}}\right]$. So, the solution of the following system is calculated by common methods in linear algebra:

(32) $$\begin{array}{c} \left[\begin{array}{cccccc} -2 & 4 & -3 & -5 & 0 & -2\\ 0 & 4 & -6 & -2 & 6 & -4\\ 5 & 0 & -2 & -3 & 8 & 3\\ -9 & 1 & 8 & -8 & 4 & 7 \end{array}\right]\left[\begin{array}{c} M_{\tilde{X_{1}}}\\ M_{\tilde{X_{2}}}\\ M_{\tilde{X_{3}}}\\ M_{\tilde{X_{4}}}\\ M_{\tilde{X_{5}}}\\ M_{\tilde{X_{6}}} \end{array}\right]=\left[\begin{array}{c} 0\\ 6\\ -3\\ 2.5 \end{array}\right]. \end{array}$$

Here right hand side is $M_{\text{Supp}(\tilde{B})}$ and $M_{\tilde{X_{j}}}=\left(\bar{x_{j}}+\underline{x_{j}}\right)/2$. Therefore, the solution of the center of $\left[\underline{x_{j}},\bar{x_{j}}\right]$ is as follows:

(33) $$\begin{array}{c} M_{X}={\left[\begin{array}{cccccc} -1.37315, & 0, & -0.63725, & 0.9316, & 0.67325, & 0 \end{array}\right]}^{t}. \end{array}$$

Finally, a solution for $\text{Supp}\;(\tilde{X})$ is as follows according to Theorem 10 and solutions (32) and (33):

(34) Supp(X~)=[SX1~SX2~SX3~SX4~SX5~SX6~]=[[x1_,x1¯][x2_,x2¯][x3_,x3¯][x4_,x4¯][x5_,x5¯][x6_,x6¯]]=[[−1.4063,−1.3400][−0.0697,0.0697][−0.6920,−0.5825][0.8602,1.0030][0.5095,0.8370][−0.0668,0.0668]].

Now we come back to the second stage. According to Theorem 13, solving system (28) is equivalent to the presence of a solution for the following system:

(35) Z∗=min⁡ xa1+xa2+xa3+xa4s.t.[−24−3−50−204−6−26−450−2−383−918−847][x1x2x3x4x5x6] +[xa1xa2xa3xa4]=[05−32],   xj_≤xj≤xj¯ j=1,2,…,6,0≤xai~ i=1,…,4.

Again, we solve this problem with the* Simplex* method. Since the optimal value is zero, system (28) has the following solution:

(36) $$\begin{array}{c} \text{Core}\left(\tilde{X}\right)=\left[\begin{array}{c} x_{1}\\ x_{2}\\ x_{3}\\ x_{4}\\ x_{5}\\ x_{6} \end{array}\right]=\left[\begin{array}{c} -1.3400\\ -0.0697\\ -0.6920\\ 0.8707\\ 0.5193\\ 0.0620 \end{array}\right]. \end{array}$$

According to Theorem 12 and solutions (34) and (38) a final solution for system (26) is as follows:

(37) X~=[(x1_,x1,x1¯)(x2_,x2,x2¯)(x3_,x3,x3¯)(x4_,x4,x4¯)(x5_,x5,x5¯)(x6_,x6,x6¯)]=[(−1.4063,−1.3400,−1.3400)(−0.0697,−0.0697,0.0697)(−0.6920,−0.6920,−0.5825)(0.8602,0.8707,1.0030)(0.5095,0.5193,0.8370)(−0.0668,0.0620,0.0668)].

In this example, since different solutions can be obtained in the first or second stage (e.g., in the second substage), other solutions can also be achieved. This is not possible in numerical methods [2–5] or the embedding method [1].

**Example 2 .**

Consider the 4 × 6 interval system:

(38) 6x1−6x2−2x38x4−2x5=[−2,0]−5x1−4x2+6x3−4x4+x55x6=[3,6]8x1+2x2+x3+5x4−8x58x6=[2,3]−3x1+5x4−8x5−7x6=[0,5].

Here system (39) is solved. It is an interval system, so it is solved in two stages according to Theorem 10. The first stage is to find the interval length, that is, *L*_*X*_. Thus, the following system is solved:

(39) $$\begin{array}{c} A^{+}L_{X}=L_{b}, \end{array}$$

such that

(40) $$\begin{array}{c} A^{+}=\left[\begin{array}{cccccc} 6 & 6 & 2 & 8 & 2 & 0\\ 5 & 4 & 6 & 4 & 1 & 5\\ 8 & 2 & 1 & 5 & 8 & 8\\ 3 & 0 & 0 & 5 & 8 & 4 \end{array}\right],\;\;L_{b}=\left[\begin{array}{c} 2\\ 3\\ 1\\ 5 \end{array}\right]. \end{array}$$

However, according to Theorem 11, the presence of solution (39) is equivalent to the following LP problem:

(41) Z∗=Min⁡ 1xa s.t.A+LX+xa=Lb ŁX,xa≥0.

We solve it with the* Simplex* method. Since the optimal value is not zero, in fact Z* = 2.794, system (39) has no solution. As a result, system (38) has no solution.

## 5. Conclusion

In this paper, we presented a method which is novel for transformed fuzzy system of linear equations. The proposed method is applicable rather than other existing methods. Because the base of this method is linear programming, it can explicitly express the presence or the absence of a solution. In addition, if a solution exists, it expresses the possibility of other solutions. This is not possible in numerical methods based on the embedding method. With a slight change, this method can be used for systems whose right hand side is trapezoidal fuzzy numbers; in previous methods, they did not have this ability. Finding the optimal solutions of fuzzy LP problems is one important application of solving fuzzy linear systems. With this new method, these problems can easily be solved. The presented method can introduce fundamental change in operation research with interval data. It also amends some application of linear programming with fuzzy data as some method in [12–15]. In the numerical solution of fuzzy differential equations, this method provides an explicit method so it is extremely efficient. For example, in three-diagonal matrices, parallel processing techniques can be applied on this method if the number of equations is too high. This method can analytically present the algebraic structure of fuzzy polyhedrons in the same way the representation theorem provides the algebraic structure for crisp polyhedrons. The previous methods are lacking this capability.
